# Four Pathways Involving Innate Immunity in the Pathogenesis of Preeclampsia

**DOI:** 10.3389/fcvm.2015.00020

**Published:** 2015-04-28

**Authors:** Kelsey R. Bounds, M. Karen Newell-Rogers, Brett M. Mitchell

**Affiliations:** ^1^Department of Medical Physiology, Texas A&M Health Science Center, Temple, TX, USA; ^2^Department of Surgery, Texas A&M Health Science Center, Temple, TX, USA

**Keywords:** innate immunity, preeclampsia, endothelial dysfunction, placental dysfunction, immune cells

## Abstract

The maternal innate immune system plays an important role both in normal pregnancy as well as hypertensive disorders of pregnancy including preeclampsia (PE). We propose four pathways that involve excessive innate immunity that lead to most forms of PE. Pre-existing endothelial dysfunction plus pregnancy leads to an excessive innate immune response resulting in widespread inflammation, placental and renal dysfunction, vasoconstriction, and PE. Placental dysfunction due to shallow trophoblast invasion, inadequate spiral artery remodeling, and/or low placental perfusion initiates an innate immune response leading to excessive inflammation, endothelial and renal dysfunction, and PE. A heightened innate immune system due to pre-existing or acquired infections plus the presence of a paternally derived placenta and semi-allogeneic fetus cause an excessive innate immune response which manifests as PE. Lastly, an abnormal and excessive maternal immune response to pregnancy leads to widespread inflammation, organ dysfunction, and PE. We discuss the potential role of innate immunity in each of these scenarios, as well as the overlap, and how targeting the innate immune system might lead to therapies for the treatment of PE.

## Preeclampsia and Innate Immunity

Preeclampsia (PE) is a syndrome in which hypertension and proteinuria or end-organ damage develops during pregnancy. PE, which affects 5–8% of all pregnancies, usually manifests as early onset (<34 weeks) or late onset (≥34 weeks) and is the leading cause of preterm births. While PE typically resolves post-partum, there is a strong connection between PE and an increased risk of developing cardiovascular disease later in life. In pretty much all studies of women with PE, there are increased levels of pro-inflammatory immune cells and cytokines, decreased levels of regulatory immune cells and cytokines, and/or the ratio of pro-inflammatory to anti-inflammatory immune cells and cytokines is increased. This strongly implicates the maternal immune system as a major contributor to the pathogenesis of PE; however, whether excessive activation of the maternal immune system initiates the development of PE or participates at a later stage in PE or both is unclear.

The maternal innate immune system acts as both a protector and effector during pregnancy. As protector, innate immune cells including macrophages, dendritic cells, natural killer (NK) cells, neutrophils, and γδ T cells are upregulated during pregnancy in order to protect the mother from pathogens while her adaptive immune system is dampened so as not to elicit a specific immune response toward the fetus. As effector during normal pregnancy, innate immune cells are important in blastocyst implantation, placentation, trophoblast invasion, and spiral artery remodeling and the cellular regulation and repair of the tissues involved in these processes. They are also involved in fetal tolerance throughout pregnancy as well as parturition. However, innate immune cells can switch from a tolerogenic, anti-inflammatory phenotype to a cytotoxic, pro-inflammatory phenotype upon the sensing of pathogens or endogenous danger signals such as RNA, DNA, heat shock proteins, uric acid, tumor necrosis factor, etc. via their expression of highly conserved pattern recognition receptors. As cytotoxic effector cells, innate immune cells create a state of oxidative stress by releasing reactive oxygen/nitrogen species (ROS/RNS), inflammation via cytokine release and activation of adaptive T and B cells, and placental ischemia via reduced angiogenesis and increased vasoconstriction, in an attempt to cause cell death, fibrosis, and rejection of the fetus. We believe that the timing and level of innate immune system activation during pregnancy corresponds to not only the severity of PE but also pregnancy outcome.

Does excessive activation of the maternal innate immune system initiate the development of PE, participate at a later stage, or both? We believe the answer is both. In this review, we present evidence that excessive innate immunity during pregnancy can lead to the clinical manifestations of PE regardless of the initiating pathophysiology which can be divided into four pathways.

## Four Pathways Involving Innate Immunity in the Pathogenesis of Preeclampsia

**Figure 1:**
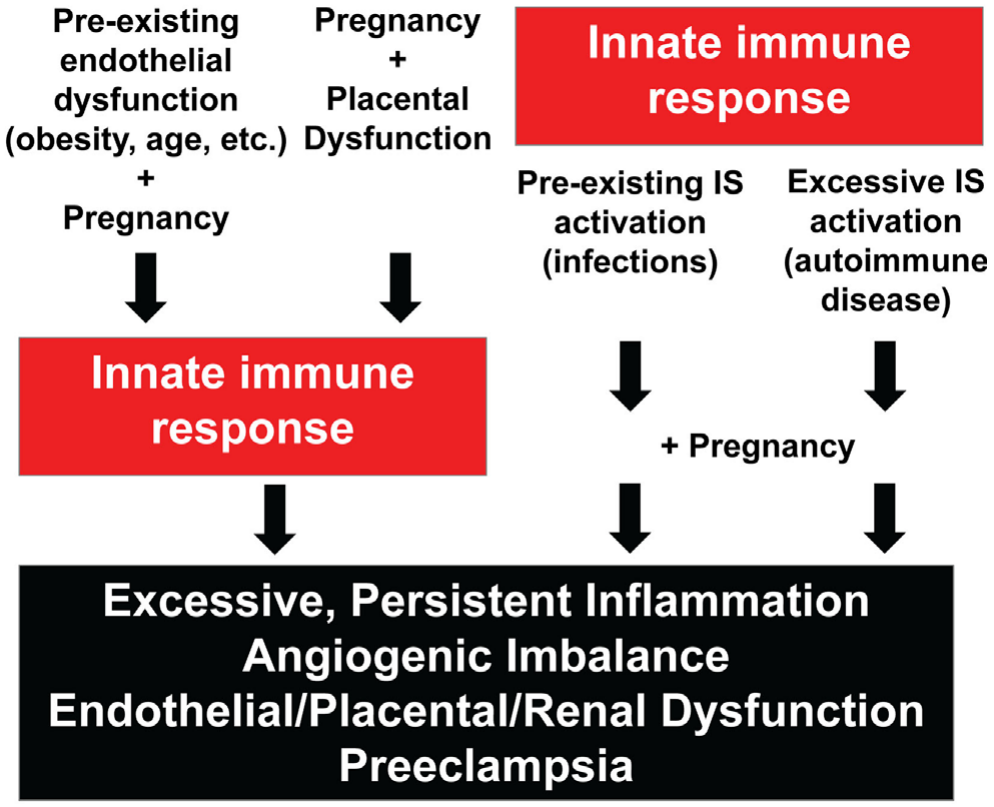
**Four pathways involving innate immunity in the pathogenesis of preeclampsia**. IS, immune system.

Based on known risk factors for the development of PE as well as experimental and circumstantial clinical evidence, we propose four pathways involving excessive innate immune system activation that can lead to PE. Two pathways consist of excessive innate immunity in response to abnormal physiology which in turn leads to inflammation, angiogenic imbalance, endothelial/placental/renal dysfunction, and PE. The other two pathways consist of heightened or abnormal innate immunity prior to and/or during pregnancy leading to excessive inflammation, angiogenic imbalance, endothelial/placental/renal dysfunction, and PE. The four pathways are illustrated in Figure 1.

## Endothelial Dysfunction, Pregnancy, and Innate Immunity

The first pathway involves pre-existing endothelial dysfunction that, when coupled with the adaptations of pregnancy, is not sufficient to provide blood flow to the appropriate tissues. Pregnancy is considered a physiological stress test and the low-grade inflammation evident in the vasculature of obese women, women >35 years of age, women with either diabetes mellitus type I or II (T1DM and T2DM), and hyperlipidemia starts the mother off in a predicament. The increased volume, cellular metabolic activity, and tissue demands of pregnancy all strain the dysfunctional endothelium of the maternal systemic and reproductive vasculature and elicit further damage leading to enhanced activation and recruitment of innate immune cells and an augmented inflammatory immune response.

Obesity is a known risk factor for PE, but the mechanisms are not yet known. Bodnar and colleagues studied the occurrence of PE in women who were divided into groups based on their body mass index (BMI) and they found a correlation between high pregnancy BMI and risk for both mild and severe PE (1). This corroborates another study that searched for different risk factors associated with severe PE. Stone et al. retrospectively compared women with PE versus normotensive pregnant women and found that the only risk factors related to severe PE were a history of the disease and severe maternal obesity (2). A question that arises is whether the pregnant women have a high BMI before becoming pregnant or gained the weight during pregnancy and whether or not that has an impact on the development of PE. Increased pre-pregnancy BMI was found to be significantly associated with an increase in the risk for PE in a population-based cohort study done by Ros and colleagues (3). They found that 9.1% of overweight (BMI = 26.1–29.0) and 12.9% of obese (BMI > 29.0) women developed PE compared to 3.1% of underweight (BMI < 19.8) and 4.5% of normal weight (BMI = 19.8–26.0) women. The women in the obese category had an odds ratio of 5.19 for PE. Another study done by Bianco et al. looked at morbidly obese (BMI > 35) pregnant women and found that these women were more likely to develop PE compared to non-obese women (4). They also reported that gestational weight gain did not affect the risk of developing PE. Saftlas et al. studied the difference between pre-pregnancy BMI and gestational weight gain and whether either one caused an increased risk of developing PE (5). They concluded that women who were considered obese before pregnancy had a higher risk of PE. In contrast, women who gained more weight than expected during pregnancy did not have an increased risk for PE; however, they did have a higher risk of developing transient hypertension. Gestational weight gain does not seem to contribute much to the risk of developing PE compared to pre-pregnancy obesity, as it is not always associated with an increased BMI and thus is not tightly linked to PE (6). Morbid obesity is highly associated with endothelial dysfunction. Mauricio and colleagues stated that there are many contributions to endothelial dysfunction in obese patients, but the most outstanding factors included diminished bioavailability of nitric oxide, oxidative stress, chronic inflammation, increased amounts of vasoconstrictors, and decreased amounts of vasodilators in the body (7). Women with a higher BMI begin pregnancy with an increased blood volume, cardiac output, and blood pressure as well as chronic inflammation and endothelial dysfunction which in turn may cause PE when these become exacerbated during pregnancy. We propose that this occurs when the innate immune system is excessively activated by danger signals stemming from the recurring endothelial injury and dysfunction. Innate immune system activation then augments the inflammation, oxidative stress, and vasoconstriction which manifests as PE. Experimental evidence supports this contention as a loss of nitric oxide, vascular endothelial growth factor (VEGF), or transforming growth factor beta (TGFβ) bioavailability during pregnancy as well as ingesting a high-fat diet can induce PE-like features in animals (8–11).

Several studies have reported a correlation between obesity and neutrophil infiltration of the systemic vasculature in pregnant women. Neutrophils help protect the host from infection by producing ROS/RNS as well as proteolytic enzymes that are helpful in fighting infection, but that can sometimes be toxic to the host tissue as a part of a “by-stander” type injury. Leik and Walsh reported that neutrophils act as carriers of oxidative stress from the placenta to the maternal vasculature by adhering to the endothelium (12). They biopsied highly vascularized subcutaneous fat at the time of cesarean section from normal pregnant and women with PE and stained for interleukin (IL)-8, intercellular adhesion molecule-1 (ICAM-1), and cluster of differentiation 66b (CD66b). There was a greater amount of staining for IL-8, an inflammatory marker, observed in the vascular smooth muscle and endothelial layers of women with PE. This represented the migration of neutrophils from the circulation to the vascular smooth muscles because neutrophils tend to travel on a concentration gradient toward increasing concentrations of IL-8. ICAM-1 was found on the endothelium in all groups, but only in the vascular smooth muscle layer of the PE group which supports the idea that inflammation affects the vascular smooth muscle cells of women with PE. Levels of CD66b were significantly greater in women with PE suggesting that there were more neutrophils present. There were also more vessels containing adhered and flattened neutrophils on the endothelium in women with PE. This connection between neutrophils and inflammation could be one of the links between obesity, innate immunity, and PE.

Overweight and obese women are more likely to be diagnosed with chronic disease risk factors such as hypercholesterolemia which further complicates their pregnancies. Elevated levels of triglycerides (TG) and low density lipoproteins (LDL) are present in many women with PE suggesting that women with hyperlipidemia may be at risk for developing PE. The main focus of concern is high levels of TGs in the body. Hubel et al. found that women with PE had significantly higher amounts of TG in their serum, but the high density lipoproteins (HDL) and LDL concentrations did not differ between women with PE and normal pregnant women (13). Sattar et al. produced similar findings and both groups suggest that the oxidation of TGs play a role in the development of endothelial dysfunction in women with PE (14). According to Granger et al., the significant increase in TG in women with PE correlates with an increase in small dense LDLs (15). Fatty acids serve as substrates for lipid peroxidation which is also significantly increased in women with PE. ApoC3 transgenic mice, which exhibit abnormal fatty acid metabolism, display PE-like features (16–18). Oxidation of lipids in the body can directly cause endothelial dysfunction as well as act as danger signals that activate the innate immune system which further worsens endothelial injury. Xu and colleagues found the pro-inflammatory innate immune system receptor TLR4 in lipid-rich, macrophage-infiltrated atherosclerotic areas of humans and apolipoprotein E deficient mice (19). They also discovered that TLR4 is upregulated by oxidized LDL in cultured macrophages. Tuten et al. investigated the relationship between polymorphisms in lectin-like oxidized low-density lipoprotein receptor (LOX-1) genes and circulating sLOX-1 and oxLDL levels and the risk of PE (20). They found that certain polymorphisms of the LOX-1 gene (LOX-1 3′UTR188C>T and K167N) and high plasma levels of sLOX-1 were significantly associated with an increased risk of developing PE. Sankaralingam et al. reported an upregulation of LOX-1 and arginase in the vasculature of women with PE which can contribute to oxidative stress (21, 22). The mechanism of how this occurs is still unclear. However, they found that methylglyoxal, which is involved in vascular complications of diabetes mellitus and the development of hypertension, is a possible factor that affects LOX-1 and arginase because it is able to induce oxidative stress in vascular cells. Another finding by Zhang and colleagues suggests that LOX-1 accumulation may contribute to the development of PE by promoting sFlt-1 production in trophoblasts (23). This group was able to inhibit LOX-1 and protect against oxidative stress-mediated trophoblast dysfunction. We believe that hyperlipidemia, excessive lipid oxidation, and endothelial dysfunction coupled with a persistent innate immune response during pregnancy can elicit PE in some women.

A chronic disease that can result from obesity and hyperlipidemia is T2DM. T2DM is characterized by insulin resistance which is usually intensified by pregnancy thus complicating maternal health. An outcome analysis of pregnancies in women with T2DM showed that diabetic women were diagnosed with PE two times more than non-diabetic women (24). Garner et al. performed a prospective controlled study comparing the incidence of PE and maternal–fetal outcome in diabetic pregnancies and non-diabetic pregnancies (25). Diabetic women were 9.9% more likely to develop PE compared to 4.3% in non-diabetic pregnant women. PE also became more prevalent with the increasing severity of diabetes. One might ask whether there is a difference between the prevalence of PE in T1DM and T2DM. Cundy et al. compared pregnant women with T1DM and T2DM and found that the incidence of hypertension was similar, but the subgroups of hypertension were different (26). Women with T2DM were more inclined to have chronic hypertension whereas women with T1DM were more frequently diagnosed with PE. Up to 39% of pregnant women with T1DM are affected by PE. This could be explained by the hyperglycemia causing endothelial dysfunction in the maternal and placental vessels prompting innate immunity and the development of PE (27, 28). There are many speculations as to how hyperglycemia affects endothelial cell function, but the ideas that are most prevalent are that hyperglycemia causes the activation of the polyol pathway, the activation of protein kinase C, and increases oxidative stress (29). These different pathways seem to overlap in certain aspects so they are considered to work together in some ways to diminish endothelial cell function. Hyperglycemic patients also tend to exhibit increased innate immunity causing inflammation. Liu et al. examined the effects of high glucose on macrophages and found that proliferation increased with greater concentrations of glucose possibly due to increased CSF-1 receptor expression (30). Increased macrophages and macrophage polarization to pro-inflammatory M1 cells result in inflammation because of their innate immune system activity (31). The deteriorating endothelial dysfunction and innate immune system activation due to hyperglycemia may be sufficient in some women to cause PE.

Advanced maternal age (AMA; 35 years or older) is considered another risk factor for PE. In the United States, the risk of PE increases by 30% every year beyond 34 (32). Little is known as to why PE occurs more often in older pregnant women but there has been some speculation. Bianco and colleagues found that out of 1,404 pregnant women, older gravidas were more likely to develop PE which correlated with other serious pregnancy related diseases (33). Lee et al. studied the prevalence of PE in an Asian population and found that women above the age of 34 years were more likely to develop PE (34). Dorjgochoo and colleagues measured *in vivo* lipid peroxidation and found that levels of a biomarker significantly increased with age in middle-aged and postmenopausal women (35). They also found that a marker for oxidative stress was positively associated with age. Csiszar and colleagues suggest that age-related oxidative stress may promote vascular inflammation and endothelial dysfunction (36). Sorescu et al. found that increased expression of NOX4/NADPH oxidase (ROS producing enzyme) correlated positively with vascular superoxide production and atherosclerosis and inflammation in aging humans (37). There is also emerging data suggesting that the innate immune system is upregulated during aging (38). This supports the notion that the innate immune system is involved in inflammation, endothelial dysfunction, and PE during AMA pregnancy.

## Pregnancy, Placental Dysfunction, and Innate Immunity

The second pathway involves placental dysfunction leading to innate immune system activation and resulting in inflammation, endothelial/renal dysfunction, and PE. Danger signals including RNA, DNA, heat shock proteins, uric acid, tumor necrosis factor, and others released from the placenta “tell” the mother that the placenta either did not form properly or is not functioning adequately (39). This would lead to fetal rejection as the mother attempts to terminate the pregnancy and save herself. As in solid organ transplant rejection, the result of innate immune system activation toward an organ results in innate and adaptive immune cell infiltration, inflammation, decreased angiogenesis, and reduced perfusion in an effort to cause ischemia, fibrosis, and cell death. It has been suggested that the severity of PE is associated with how strong or weak the innate immune response is.

Placental dysfunction including shallow trophoblast invasion, deficient spiral artery remodeling, and low placental perfusion are known to be involved in the development of PE; however, the root of this problem is still unknown (40, 41). In normal pregnancy, blastocysts latch on to the maternal decidua then the cytotrophoblast cells (CTBs) proliferate and create extravillous trophoblasts on the very tip of their columns. These cells invade the decidua and differentiate into either endovascular or interstitial trophoblasts. While the interstitial trophoblasts embed themselves into the inner myometrium, the endovascular trophoblasts, derived from the male, start migrating toward the maternal spiral arteries. At around 10–12 weeks of gestation in women the trophoblasts break down the maternal vessel walls which create low-resistance vessels to maintain sufficient placental perfusion. When this process does not progress successfully, PE has been shown to develop. This means that the maternal innate immune system has to allow the perfect interaction between the paternally derived trophoblasts and the maternal tissue for proper invasion. Different studies have looked into the cause of trophoblast invasion failure.

Zhou et al. suggests that in normal pregnancies, in order for the CTBs to invade the uterine interstitium and vasculature the CTBs have to alter their adhesion receptor phenotype to model the maternal endothelial cells they are to replace (42). In PE pregnancies this modification is thought to not occur. CTBs from normal pregnant women and CTBs from women with PE were examined for whether or not they expressed vascular cell adhesion molecule (VCAM-1) and platelet-endothelial cell adhesion molecule (PECAM-1) along with other integrins and cadherins that are characteristic of endothelial cells and certain leukocytes. They found that VCAM-1 was not found on the villous CTBs but on the CTBs within the uterine wall of normal pregnant women. PECAM-1 was expressed on the interstitial and endovascular CTBs in normal pregnant women; however, neither VCAM-1 nor PECAM-1 were found on CTBs in women with PE. Coukos et al. reported that PECAM-1 is present in the trophoblast-endothelium interaction which suggests that PECAM-1 is an important part of proper trophoblast invasion found in normal pregnancies (43). They found that certain trophoblasts express PECAM-1, suggesting that there is a subpopulation that is required for the endovascular differentiation pathway.

If this trophoblast invasion does not occur properly then danger signals are released from the placenta which are detected by the local and maternal systemic innate immune system and elicit a response from innate immune cells including macrophages, dendritic cells, NK cells, neutrophils, and γδT cells (39). Once these cells are activated excessive inflammation leads to endothelial dysfunction and PE. We and others have reported that the innate immune system receptors TLR3, TLR4, TLR7, and TLR8 are increased significantly in placentas of women with PE (44, 45). Additionally, TLR9 activation by mitochondrial DNA/fetal DNA or their mimetics during pregnancy in rodents can also cause detrimental pregnancy outcomes (46, 47). This supports the notion that when trophoblast invasion fails danger signals are released that activate the innate immune system.

If placental trophoblasts invade correctly but the maternal spiral arteries do not remodel in a sufficient manner there is also a chance of developing PE. Normal pregnancies are characterized by the formation of large arterio-venous shunts whereas during PE there are minimal shunts resulting in narrower uterine arteries (48). For a normal pregnancy to occur, it is essential that the spiral arteries adapt to the placental invasion by dilating the distal segment to ensure the delivery of large amounts of blood to the placenta at an appropriate rate and pressure. When there is improper remodeling the spiral arteries are constricted which results in faster output of blood causing the rupturing of anchoring villi as well as the dislodging of trophoblastic micro-particulate debris from the villous surface leading to maternal endothelial and immune cell activation. Finally, a reduction in blood transit time impairs oxygen exchange. During normal spiral artery remodeling, the surrounding smooth muscle loses its elasticity, but in PE the smooth muscle constricts thus reducing blood supply to the placenta. Singh and colleagues reported that pregnant mice lacking complement component C1q, necessary for trophoblast invasion and spiral artery remodeling, exhibit many features of PE supporting a role for innate immunity in placentation that, when deficient, can lead to low placental perfusion, innate immune system activation, inflammation, angiogenic imbalance, endothelial/renal dysfunction, and PE (49). In severe cases, the immunity, inflammation, angiogenic imbalance, and endothelial dysfunction can cause fibrosis of the uterine wall leading to fetal rejection.

## Infections, Innate Immunity, and Pregnancy

The third pathway involves innate immune system activation in response to infections acquired before or during pregnancy coupled with the higher sensitivity of the innate immune system during pregnancy. We propose that this “double hit” immune response exceeds a certain threshold in some women and the excessive pro-inflammatory and oxidative state then affects the vasculature, placenta, and kidneys. It has been reported that bacterial and viral infections significantly increase the risk of developing PE (50). Chronic subclinical infections are suggested to be a probable cause of inflammation in women that develop PE and that early treatment of vaginal and urinary infections reduces the incidence of developing PE (51). Another study corroborated these findings reporting that not only urinary tract infections are associated with an increased risk of PE, but also periodontal infections as well (52).

Highly conserved pattern recognition receptors including TLRs, NOD-like receptors (NLRs), and RIG-like receptors (RLRs) detect various parts of bacteria and upon receptor activation induce an innate immune response. For TLRs, bacterial lipoproteins are detected by TLR1, bacterial peptidoclycans and lipoteichoic acid of Gram-positive bacteria are detected by TLR2, heat shock proteins and lipopolysaccharide of Gram-negative bacteria are detected by TLR4, bacterial flagellin is detected by TLR5, mycoplasma is detected by TLR6, unmethylated CpG DNA is detected by TLR9, and a specific bacterial ribosomal RNA sequence is detected by TLR13, all of which reside on the surface of maternal immune and non-immune cells as well as cells in the uteroplacental unit. Activation of these TLRs leads to a rapid anti-bacterial immune response for pathogen clearance followed by adaptive immune system activation. While direct cause-and-effect studies in women showing that bacterial infections induce PE are obviously lacking, there is evidence that bacterial infections are highly associated with the development of PE and experimentally can elicit PE in rodents.

Amarasekara and colleagues reported that 12.7% of placentas from women with PE were positive for bacteria whereas all placentas from normotensive women were negative (53). Bacteria found to be present in the placentas of women with PE included *Bacillus cereus, Listeria, Salmonella, Escherichia, Klebsiella pneumonia, Anoxybacillus, Variovorax, Prevotella, Porphyromonas*, and *Dialister*. Periodontal infections from *Porphyromonas gingivalis*, *Eikenella corrodens*, and *Micromonas micros* were also found more often in women with PE (51, 54). Additionally, placental TLR4 expression is increased in women with PE compared to normotensive women (55, 56). While not all studies report a significant association between bacterial infection and PE, many do and further suggest that inflammation from chronic subclinical infection rather than acute infection or reinfection is associated with the development of PE (50). Experimentally, endotoxin/LPS given to pregnant rats and mice were able to induce PE-like features by inducing excessive inflammation (16, 57). Additionally, TLR9 activation by CpG oligonucleotides during pregnancy in rats can also elicit PE-like features (47). While these animal studies examined the effects of acute bacterial infection on pregnancy, studies in which animals are chronically infected prior to pregnancy are needed. Overall, these findings support the notion that bacterial infection and subsequent innate immune receptor activation may lead to excessive inflammation and PE in some women.

Other TLRs, NLRs, and RLRs detect viruses and upon ligation also induce an innate immune response. For TLRs, maternal and uteroplacental cells contain TLR3 which recognizes dsRNA and TLR7/8 which recognizes ssRNA in addition to TLR9 and TLR13 which also recognize patterns of DNA viruses. Activation of these TLRs induces a rapid anti-viral immune response mainly mediated by type I interferons and also leads to adaptive immune system activation. Like bacterial infections, there is circumstantial evidence associating viral infections and PE. These include adeno-associated-virus-2, parvovirus, cytomegalovirus, and herpes simplex virus. Other viruses that may be associated with PE include rhinovirus, reovirus, enterovirus, coxsackievirus, rhabdovirus, paramyxovirus, orthomyxovirus, and picornavirus. We have reported that TLR3, TLR7, and TLR8 immunoreactivity is increased in placentas of women with PE compared to normotensive women along with markedly increased staining for placental dsRNA (44). We have also discovered that maternal innate immune system activation using TLR3 or TLR7/8 agonists induces PE-like features in rats and mice and that treatment with the immunoregulatory cytokines IL-10 and IL-4 can prevent these effects (44, 58–62). Supportive of this innate immunity-PE pathway is that the induction of PE-like features by viral mimetics in rodents is pregnancy-dependent and is associated with increased pro-inflammatory immune cells and cytokines and excessive inflammation. As mentioned previously, TLR9 activation also induces PE-like features in rats (47). Future studies examining whether these various pathogens and others can directly induce PE in animals as well as testing for these pathogens early in pregnant women will help define the connection between infections and the development of PE.

## Abnormal Innate Immunity to Pregnancy

The fourth pathway involves an exaggerated maternal immune activation relative to a normal pregnancy. Supportive of this mechanism are the findings that women with a personal or familial history of autoimmune disease are at a significantly higher risk of developing PE. Each person has individualized immune responses as determined by the amount and type of major histocompatibility complex (MHC) molecules expressed on their cells. Women with a hyperactive innate immune system would initiate an exaggerated immune response to the cellular necrosis that occurs during implantation and placentation as well as to the fetal and paternal antigens. This typically manifests not only as excessive inflammation but also eventually by the presence of autoantibodies against various proteins contributing to the development of PE.

Classical studies showing that reduced exposure to paternal antigens prior to pregnancy or *in vitro* fertilization increases the risk of PE support the notion that maternal tolerance to the fetus is necessary for a normotensive, successful pregnancy. Maternal and placental immune cells receiving signals released from the fetoplacental unit usually achieve this state of tolerance; however, if maternal innate immune cells “over-ride” these signals and do not lead to a state conducive to tolerance, then excessive innate immune system activation and inflammation occur. This is what likely happens in women with autoimmune diseases. Women with systemic lupus erythematosus (SLE), rheumatoid arthritis (RA), antiphospholipid syndrome, autoimmune thyroid disease, and T1DM all have an increased risk of developing PE (25, 63, 64). The increased innate immunity in these women could be due to altered complement regulatory proteins or in the expression of placental sialic acid (64, 65). Some studies have identified that the KIR AA genotype of uterine NK cells inhibits fetal HLA-C2 expressed on trophoblasts, while genetic variants in fetal HLA-G do not dampen maternal immune activation that is associated with PE (66, 67). Lastly, decreased levels of inducible regulatory T cells (iTregs) or the ratio of iTregs to pro-inflammatory T cells have been reported to be decreased in women with autoimmune diseases as well as in women with PE (41, 68–72). iTregs are known to reduce antigen-specific immune cell activation and signaling. Hsu and colleagues reported that antigen presenting cells from the decidua of normal pregnant women greatly promote the expansion of iTregs whereas antigen presenting cells from the decidua of women with PE do not (68). This lack of regulatory control of immune activation may contribute in some women to the excessive inflammation and organ dysfunction that leads to PE.

## Links between Innate Immunity and the Manifestation of Preeclampsia

Regardless of the pathway involved, excessive activation of the innate immune system may ultimately lead to the features of PE including hypertension, proteinuria, and end-organ damage. This is likely mediated by both the innate and adaptive arms of the immune system via their effects on endothelial function (73, 74). Cytotoxic molecules produced by innate immune cells including IL-17, ROS/RNS, and IFNs all have detrimental effects on vascular function and excessive levels of these molecules in the systemic and reproductive vasculature would cause vasoconstriction and lead to organ dysfunction and damage (renal, placental, and possibly neural) (75–77). Activation of adaptive immunity by innate immune cells also occurs and numerous studies have shown that vasoactive molecules produced by T and B cells decrease endothelial function and induce PE-like features in animals (78–83). At the vascular level, endothelial cell injury due to excessive inflammation and oxidative stress leads to the production, secretion, and expression of cytokines including IL-6, chemokines such as chemokine ligand 5 (CCL5), and adhesion molecules such as VCAM, ICAM, and E-selectin. Additionally, studies in women with PE and animals with experimental PE consistently demonstrate that there is increased vascular endothelin-1 production and decreased nitric oxide bioavailability which likely results from the excessive inflammation mediated by immune system activation. More studies are needed to determine the molecular mechanisms by which innate immune system activation leads to endothelial dysfunction during pregnancy.

## Pathway Interplay and Therapeutic Targets

We believe that each pathway is independent with respect to the initiating insult; however, subsequently there is considerable overlap between the pathways. For example, excessive innate immune system activity at the beginning of pregnancy would tend to inhibit trophoblast invasion, spiral artery remodeling, and fetal tolerance which would then exacerbate innate immune system activity. Likewise, pre-existing endothelial dysfunction would have similar effects on placental development leading to innate immunity and PE. Experimentally there is evidence that one can induce PE-like features in animals through any of the four pathways, including inducing endothelial dysfunction by inhibiting NOS/VEGF/TGFβ or high-fat feeding, inducing shallow trophoblast invasion and defective placentation, or excessively activating the maternal innate immune system via bacterial or viral mimetics. However, trying to identify the initial insult in women proves tougher. Current clinical testing and technologies likely miss the initial insult but rather examine the spectrum of downstream and convergent effects following innate immune system activation which differs between women. Improving endothelial function prior to pregnancy, early imaging of placental development, treatment of infections before pregnancy or early in pregnancy, and control of autoimmune diseases before and during pregnancy may all be beneficial in reducing the incidence and severity of PE.

Given the potential central role of innate immunity in all four pathways leading to PE, novel therapeutics targeting this arm of the immune system is needed. While current immunosuppressive therapies are not feasible for pregnant women, future research identifying the innate immune cells that are persistently activated in these pathways during pregnancy as well as unique characteristics of these activated cells would aid in the development of targeted therapies that eliminate just these specific cells and leave other innate immune cells alone.

## Conclusion

Based on clinical and experimental evidence to date, we propose that PE develops through four independent pathways that either involve excessive innate immune system activation in response to abnormal physiology during pregnancy or excessive innate immunity before and/or during pregnancy. The excessive innate immunity and subsequent inflammation then converge, leading to angiogenic imbalance, endothelial/placental/renal dysfunction, and PE. The timing and severity of excessive innate immunity likely determine the onset and severity of PE. Studies that explore these proposed pathways in detail are needed to definitively determine the role of innate immune system activation under the various circumstances encountered during pregnancy. Ultimately, elimination of hyper-activated innate immune cells in PE may reduce the inflammation and endothelial dysfunction and improve maternal and fetal outcomes.

## Conflict of Interest Statement

M. Karen Newell-Rogers and Brett M. Mitchell have a relationship with VG Life Sciences Inc. Kelsey R. Bounds has no conflict of interests to declare.
